# Micro-Fourier-transform infrared reflectance spectroscopy as tool for probing IgG glycosylation in COVID-19 patients

**DOI:** 10.1038/s41598-022-08156-6

**Published:** 2022-03-11

**Authors:** Carla Carolina Silva Bandeira, Karen Cristina Rolim Madureira, Meire Bocoli Rossi, Juliana Failde Gallo, Ana Paula Marques Aguirra da Silva, Vilanilse Lopes Torres, Vinicius Alves de Lima, Norival Kesper Júnior, Janete Dias Almeida, Rodrigo Melim Zerbinati, Paulo Henrique Braz-Silva, José Angelo Lauletta Lindoso, Herculano da Silva Martinho

**Affiliations:** 1 Centro de Ciências Naturais e Humanas, Universidade Federal do ABC, Santo André, SP 09210-580 Brazil; 2 Instituto de Infectologia Emilio Ribas, São Paulo, Sp 01246-900 Brazil; 3 Instituto de Medicina Tropical de São Paulo, Universidade de São Paulo, São Paulo, SP 05403-000 Brazil; 4 Departamento de Biociências e Diagnêstico, Instituto de Ciência e Tecnologia, Universidade Estadual Paulista, São José dos Campos, SP 12245-000 Brazil; 5 Faculdade de Odontologia Departamento de Estomatologia, Universidade de São Paulo, São Paulo, SP 05508-000 Brazil; 6 Departamento de Moléstias Infecciosas e Parasitárias, Faculdade de Medicina, Universidade de São Paulo, São Paulo, SP 01255-090 Brazil

**Keywords:** Optics and photonics, Other photonics, Biophotonics, Diagnostic markers

## Abstract

It has been reported that patients diagnosed with COVID-19 become critically ill primarily around the time of activation of the adaptive immune response. However the role of antibodies in the worsening of disease is not obvious. Higher titers of anti-spike immunoglobulin IgG1 associated with low fucosylation of the antibody Fc tail have been associated to excessive inflammatory response. In contrast it has been also reported that NP-, S-, RBD- specific IgA, IgG, and IgM are not associated with SARS-CoV-2 viral load, indicating that there is no obvious correlation between antibody response and viral antigen detection. In the present work the micro-Fourier-transform infrared reflectance spectroscopy (micro-FTIR) was employed to investigate blood serum samples of healthy and COVID-19-ill (mild or oligosymptomatic) individuals (82 healthcare workers volunteers in “Instituto de Infectologia Emilio Ribas”, São Paulo, Brazil). The molecular-level-sensitive, multiplexing quantitative and qualitative FTIR data probed on 1 µL of dried biofluid was compared to signal-to-cutoff index of chemiluminescent immunoassays CLIA and ELISA (IgG antibodies against SARS-CoV-2). Our main result indicated that 1702–1785 $\hbox {cm}^{-1}$ spectral window (carbonyl C=O vibration) is a spectral marker of the degree of IgG glycosylation, allowing to probe distinctive sub-populations of COVID-19 patients, depending on their degree of severity. The specificity was 87.5 % while the detection rate of true positive was 100%. The computed area under the receiver operating curve was equivalent to CLIA, ELISA and other ATR-FTIR methods ($>0.85$). In summary, overall discrimination of healthy and COVID-19 individuals and severity prediction as well could be potentially implemented using micro-FTIR reflectance spectroscopy on blood serum samples. Considering the minimal and reagent-free sample preparation procedures combined to fast (few minutes) outcome of FTIR we can state that this technology is suitable for fast screening of immune response of individuals with COVID-19. It would be an important tool in prospective studies, helping investigate the physiology of the asymptomatic, oligosymptomatic, or severe individuals and measure the extension of infection dissemination in patients.

## Introduction

Accumulating evidence suggests that a subset of patients with severe COVID-19 may have cytokine storm syndrome^1^. It has been recommended that hyperinflammation should be recognized and treated using already approved therapies with proven safety profiles to reduce the increasing mortality^1^.

It has been noticed that patients diagnosed COVID-19 become severely ill, especially at the time of activation of the adaptive immune response^2–4^. Hoepel et al.^2^ reported that antibodies play a role in disease exacerbation at the time of seroconversion. They found that the excessive inflammatory response depends on two antibody features which are specific to patients with severe COVID -19: (1) higher titers of anti-spike IgG and (2) the anti-spike IgG of patients with severe COVID -19 is inherently more pro-inflammatory due to different glycosylation, particularly low fucosylation, of the Fc tail of the antibody. Analogously Chakraborty et al.^3^ suggested that patients with severe COVID-19 have a unique serological signature, including an increased likelihood of IgG1 with afucosylated Fc glycans. On the other hand, Luo et al.^4^ showed that NP -, S-, RBD- specific IgA, IgG, and IgM were not associated with SARS-CoV-2 viral load, suggesting that there is no obvious correlation between antibody response and viral antigen detected in nasopharyngeal swabs. They analyzed antibody and cytokine responses in COVID-19 from asymptomatic to severe patients (123 serum samples from 63 COVID -19 patients) and evaluated the impact of various risk factors, including comorbidities, male sex, and advancing age on the host immune response COVID-19 patients. These antagonistic data highlight the relevance of investigating structural aspects of serum IgG from COVID-19 patients to establish its rule as a severity marker.

Vibrational spectroscopic techniques such as Fourier-transform infrared absorption (FTIR) have been successfully used to study biological samples. They provide quantitative and qualitative multiplexed information at the molecular level, even when triggered by subtle changes in a sample^5^. Vibrational spectroscopy involves label-free techniques that allow detection of electronic changes in the internal vibrational energy levels of biomolecules. It has been used in the study of cells, tissues and biofluids and provided valuable insights into pathologies^5–8^. Barauna et al.^9^ investigated saliva samples from throat swabs using Attenuated Total Reflectance FTIR (ATR-FTIR). Initial classification of swab samples into negative and positive COVID-19 infections was based on symptoms and PCR testing results($n = 111$ negatives and 70 positives). A blind sensitivity of 95% and specificity of 89% were achieved. Artificial saliva samples containing inactivated $\gamma$-irradiated COVID-19 virus particles at concentrations up to 1582 copies/mL were also analyzed to understand the spectral response of the virus^9^. Proof-of-concept study by Nogueira et al.^10^ conducted on 243 patients indicated that ATR-FTIR could be a cost-effective solution for high-throughput screening of suspect patients for COVID-19 using oropharyngeal swab suspension fluid. Sensitivity, specificity, and accuracy better than 84%, 64%, and 76.9% were found, respectively. Kitane et al.^11^ reported a method for COVID -19 discrimination based on spectral ATR-FTIR analysis of 280 RNA extracts from nasopharyngeal samples (100 SARS-CoV-2 PCR-positive patients and 180 SARS-CoV-2 PCR-negative patients). The proposed method is based on ATR-FTIR analysis of the extracted RNA and machine learning modeling. The authors report that this technique achieves 97.8% accuracy, 97% sensitivity, and 98.3% specificity while reducing the testing time from hours to minutes after RNA extraction. The reported area under the ROC curve (*AUC*) ranged from 0.54 to 1, depending on the statistical model used. Wood et al.^12^ proposed a portable infrared spectrometer with custom-made transflectance accessories for rapid point-of-care detection of COVID-19 markers in saliva. They tested the system on samples from 29 subjects who tested positive for SARS-CoV-2 by RT-PCR and 28 who were negative, and achieved a sensitivity of 93% and a specificity of 82%. However the work of Wood et al. did not aim to explore the rich biochemical information contained in the spectral data.

Some clues concerning immune response and COVID-19 had been considered by Dogan et al.^13^. They used ATR-FTIR to investigate serum samples from 56 patients and revealed that the CoronaVac-Sinovac COVID-19 vaccine administration induced significant changes in some functional groups belonging to lipids, proteins and nucleic acids. FTIR has been used to investigate the secondary structural composition and changes in structural dynamics of IgG upon glycation, oxidation and glycoxidation^14^. Thus, it is a suitable technology to study structural changes in IgG induced by COVID-19.

Aiming to investigate possible correlation between antibody response and SARS-CoV-2 infection blood serum samples from adult healthcare workers with COVID-19 (asymptomatic or mild level) and healthy individuals were investigated here by micro-FTIR reflectance spectroscopy. Detailed characterization of the immune response of asymptomatic or oligosymptomatic individuals is important for prospective studies to describe the physiology of these cases compared to cases with higher severity and also to determine the extent of spread of infection by these patients.

## Methods

### Sampled population

Serum samples from 82 healthcare workers volunteers of the “Instituto de Infectologia Emilio Ribas”, São Paulo, Brazil were included in this study. These samples are part of a cohort study to evaluate the seroprevalence of SARS-CoV-2 infection in healthcare population. Samples were collected from July to November of 2020, before the beginning of the vaccination program in Brazil. Levels of illness were mild or oligosymptomatic. This study was conducted in accordance with the Declaration of Helsinki, and the protocol was approved by Research Ethics Committee of the “Instituto de Infectologia Emílio Ribas”, São Paulo, Brazil, protocol number CAAE 32264120.5.2001.0061. Invited volunteers were informed about the objectives, propositions and conditions of this project, and those who agreed to participate in the research signed the free and informed consent term. A volume of 5 mL of whole blood was collected by peripheral vein puncture and stored in a 10 mL dry tube for each patient. Then it was centrifuged at 2052 g for 10 min to separate the serum. Two mL of serum were stored in a $-20\,^{\circ }$C freezer until performing the immunoassays and FTIR tests.

### Detection of antibodies anti-SARS-CoV-2

#### Chemiluminescent immunoassay (CLIA)

A CLIA (IgG Antibodies against SARS-CoV-2; reagents pack # 619 9919; VITROS Immunodiagnostic calibrator # 619 992; Ortho Clinical Diagnostics, Raritan, NJ, USA) immunoassay was performed to detect immunoglobulin G anti-Spike protein from SARS-CoV-2. This assay does not differentiate binding IgG antibodies from virus-neutralizing IgG antibodies^15^. The results were expressed in terms of ratio of the sample signal to a calibrator-assigned cutoff signal with threshold of 1.0. Negative diagnosis were considered for outcomes with reactivity index $< 1$ otherwise they were considered positive^16^. The test sensitivity was reported to be 90.0% ($\ge 8$ days) and specificity of 100.0%^16^. This CLIA diagnostic test uses S antigens for SARS-CoV-2 detection^17^.

#### Enzyme-linked immunosorbent assay (ELISA)

Anti-SARS-CoV-2 IgG ELISA (Euroimmun Medizinische Labordiagnostika, Lübeck, Germany; # EI 2606-9601 G; Indirect ELISA) assay was performed to detect IgG antibodies against SARS-CoV-2. In this diagnostic test IgG antibodies against SARS-CoV-2 spike protein subunit 1 (S1) are detected in human serum or plasma. Following the instructions of the manufactures, serum samples were 1 : 101 diluted, added to wells coated with recombinant SARS-CoV-2 antigen and incubated for 60 min at $37\,^{\circ }$C. Then, wells were washed three times and followed by the addition of HRP-conjugated anti-human IgG and subsequent incubation for 30 min at $37\,^{\circ }$C. Wells were washed three times and a chromogen solution was added. After 30 min of incubation at room temperature, the reaction was stopped and the absorbance at 450 nm with reference at 620 nm was read on a microplate reader. A ratio between the extinction of the sample and calibrator on each plate were calculated. According to the recommendations of the manufacturer, a signal-to-cutoff ratio smaller than 0.8 is considered negative, while a positive one if greater than 1.1. The borderline region falls into 0.8–1.1 interval. The sensitivity and specificity of this assay were reported to be 90.0% and 100%, respectively^17^.

### Fourier-transform infrared absorption (FTIR) reflectance spectroscopy

**Figure 1:**
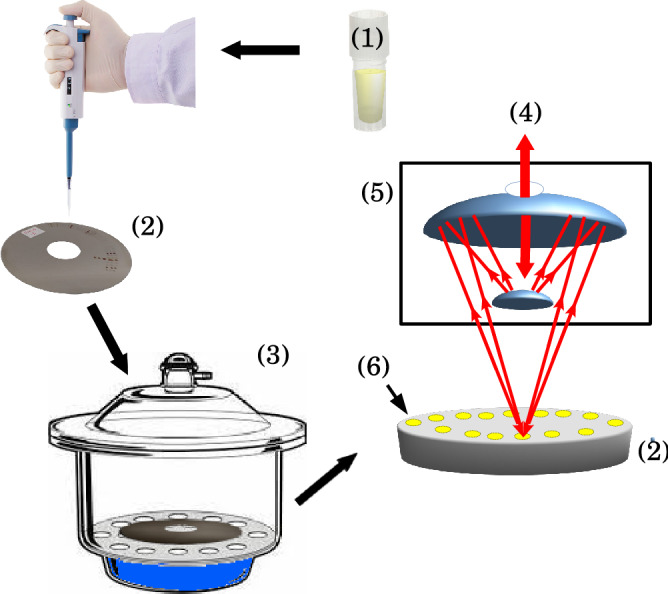
Scheme for micro-FTIR reflectance measurements for human serum. One µL of diluted (1 : 3 in ultra-pure water) serum sample solution (1) was transferred to a circular platinum sample holder (2). Then the sample holder was installed in a desiccator with saturated solution of NaCl (3) which controls the relative moisture in 80%. The drying time was 10 min. After this period the sample holder was installed on the FTIR reflectance accessory of the micro-FTIR spectrometer. The IR beam (4) passing through the IR $60\times$ magnification Cassegrain lens (5) focuses the light on a given sample (6). The reflected light is collected by the same lens and analyzed by the spectrometer.

All samples were brought to room temperature prior to preparation for micro-FTIR measurements. Aliquots of 1 µL of serum (1 : 3 in ultrapure water) were deposited in platinum sample holder and dried at 80% relative moisture under a desiccator with NaCl saturated solution in order to avoid coffee ring effect and obtain a homogeneous biofilm^18^. The final droplet presented an average radius of $1000\pm 150$ µm. A Varian 610 FT-IR micro-spectrometer equipped with a linearized MCT detector with a $100\times 100$µm detector element (InfraRed Associates, Inc.) coupled to a 640-IR FT-IR spectrometer was used in reflectance mode for spectra acquisition. After optical focusing, the microscope aperture in the Cassegrain collecting lens ($60\times$ magnification) was reduced to $150\times 150$ µm^2^ to set the area of interest for measurement on the sample. The spectral resolution was set to $2\,\hbox {cm}^{-1}$ in order to obtain the maximum throughput in the IR microspectrometer. The number of scans was 32 per sample (equivalent to 30 s time of acquisition). The scheme of methodology is shown on Fig. 1.

### Statistical analysis

#### Principal components analysis (PCA)

The classical Principal Components Analysis (PCA)^19^ was performed on mean centered raw data to extract outliers and identify possible experimental bias. All spectral analysis steps were implemented in the ChemSpec vignette available in the software R^20^. Outliers were identified using the *Q* and $T^{2}$ Hotelling’s statistics. The *Q* statistics indicates how well each observation matches to the PCA model and the $Q$-residuals measure the residual between a sample and its projection on the factors retained in the model. Large residual outliers can be detected by inspection of $Q$-residuals. On the other hand, Hotelling’s $T^{2}$ value represents a measure of the variation in each sample within the model, indicating how far each sample is from the center (scores $= 0$) of the model. It is a quantifier for scores outliers. The $T^{2}$ Hotelling’s versus $Q$-residuals (reduced) plot were inspected raw spectral data.

#### Partial least squares: discriminant analysis (PLS-DA)

All spectra were pre-processed to become comparable for statistical analysis. The baseline was corrected using the least-squares polynomial curve fitting method as described by Lieber and Mahadevan-Jansen^21^. All spectra were normalized and scaled using probabilistic quotient normalization^22^. Then PLS-DA analysis was performed. It is a multivariate supervised method that uses linear regression of original variables to predict the class membership. In our case the PLS regression was performed using the *plsr* function provided by R *pls* package^20,23^. The classification and cross-validation were performed using the corresponding wrapper function using the *caret* package^20^. A permutation test was performed to assess the performance of class discrimination. In each permutation, a PLS-DA model was built between the data and the permuted class labels using the optimal number of components determined by leave-one-out cross validation for the model based on the original class assignment. The class discrimination performance was measured using classification accuracy, $R^{2}$, and $Q^{2}$ parameters. The first one is based on prediction accuracy. The $R^{2}$ parameter is the “goodness of fit” or explained variation which is based on the ratio of the between group sum of the squares and the within group sum of squares. On the other hand, $Q^{2}$ is the ”goodness of prediction”, or predicted variation, calculated from cross validation. In each round, the predicted data are compared with the original data, and the sum of squared errors is calculated and then summed over all samples (Predicted Residual Sum of Squares or PRESS). For convenience, the PRESS is divided by the initial sum of squares and subtracted by 1 to scale to $R^{2}$. Good predictions will have low PRESS or high $Q^{2}$ while negative $Q^{2}$ means the model is not predictive at all or is overfitted^8,24,25^. Two quantifiers were used to measure the vibrational band frequency importance in PLS-DA model. The first, Variable Importance in Projection (VIP) is a weighted sum of squares of the PLS loadings taking into account the amount of explained spectral intensity-variation in each dimension. The other importance measure is based on the weighted sum of PLS regression. The weights are a function of the reduction of the sums of squares across the number of PLS components. For multiple-group analysis, the same number of predictors will be built for each group and the average of the feature coefficients were used to indicate the overall coefficient-based importance. The receiver operating characteristic (ROC) analysis was used to evaluate the discriminating performance and the area under the ROC curve (*AUC*) as its summary index. In general tests with excellent discriminating capability will furnish $AUC > 0.80$^26,27^.

#### Normality and F tests

The Kolmogorov-Smirnov test of normality^28^ was applied to test the hypothesis that IgG data does not differ significantly from that which is normally distributed. The F-test was used to test the hypothesis of equality of averages of two sets of data populations of unequal size (IgG and demographic data)^29^

**Table 1:** Demographic data of volunteers, a set of 82 healthcare workers from “Instituto de Infectologia Emilio Ribas”, São Paulo, Brazil.

Variable	Positive ($n=33$)	Negative ($n=49$)	*p*-value
Female	76.7%	72.1%	$<5$%
Male	23.3%	27.9%	$<5$%
Age	$44\pm 14$	$49\pm 12$	$<5$%
Comorbidities	39.4%	16.3%	45.4%
Body mass index (kg/m$^{2}$)	$25\pm 12$	$30\pm 12$	$<5$%

The list of comorbidities includes rheumatoid arthritis, asthma, diabetes, systemic arterial hypertension, chronic obstructive pulmonary disease, obesity, and hypothyroidism.

.

## Results and discussion

The main clinical and demographic pieces of information about volunteers are presented on Table 1. Percentages of 40.2% ($n=33$) and 59.8% ($n=49$) of individuals tested positive and negative, respectively. Female individuals predominated in the sampled groups ($>72$ % of total number of patients). However we notice that this unbalance did not induce a bias on the positive or negative rates within the confidence value of 5%. The average age of patients in positive and negative groups was $44\pm 14$ years and $49\pm 12$ years, respectively. Again these differences were not statistically relevant for COVID-19 diagnostic purposes. Likewise the body mass index did not represent a statistically relevant variable comparing negative and positive groups. On the other hand, the presence of comorbidities presented a well-defined increased risk for positive test against COVID-19. A percentage of 39.4% of individuals which tested positive presented some kind of comorbidity (rheumatoid arthritis, asthma, diabetes, systemic arterial hypertension, chronic obstructive pulmonary disease, obesity, or hypothyroidism) while this rate is only 16.3% in negative group. The outcome of F-test indicated that there is enough evidence against the hypothesis that the population sampled with positive and negative test has the same average ($p=45.4$%). The correlation between comorbidities and prevalence of COVID-19 is well reported in literature and our findings give one more piece of evidence concerning this important aspect of etiology of COVID-19^30^. We notice that CLIA and ELISA tests were 100% concordant about the patient’s diagnosis in our cohort.

**Figure 2:**
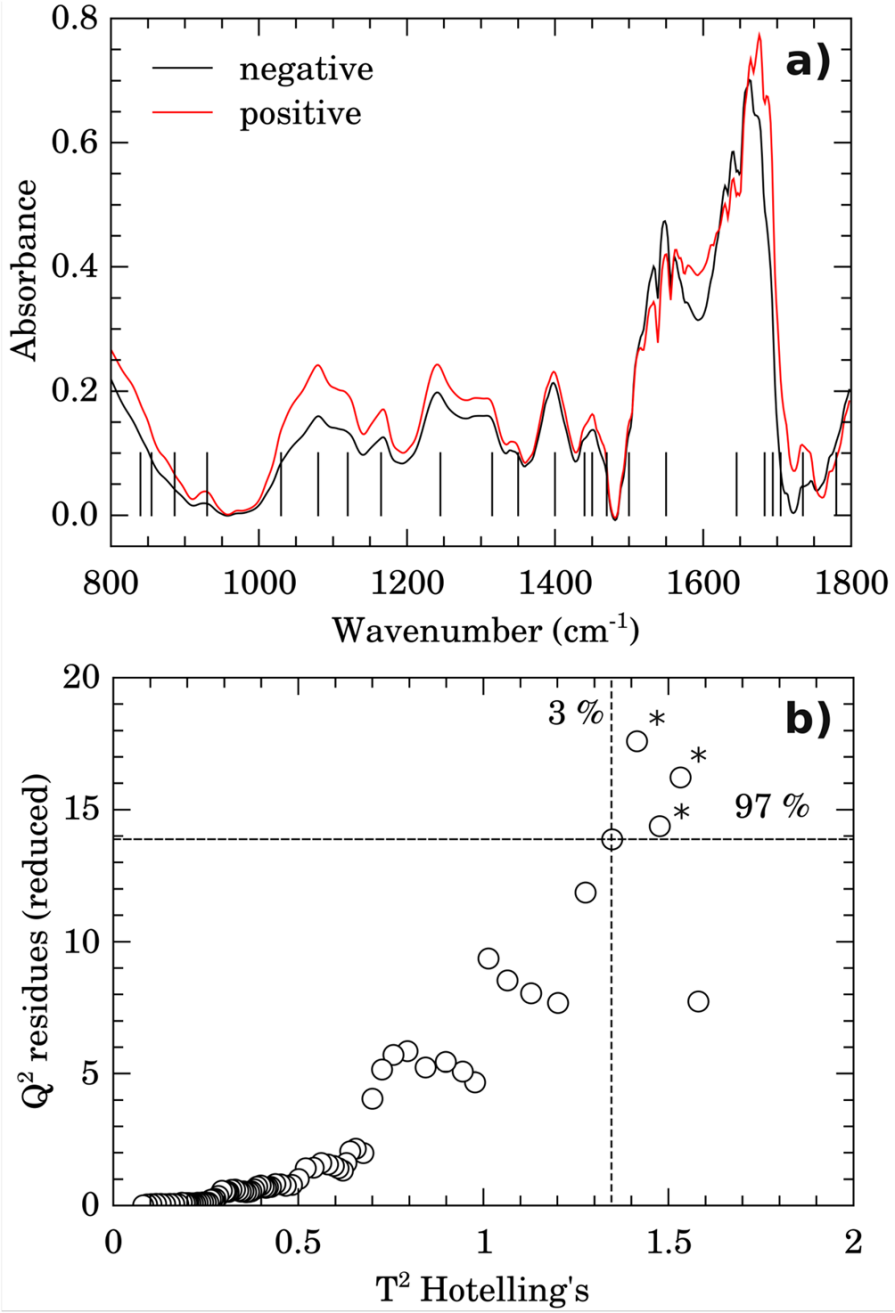
Average spectra and outliers. **(a)** Average micro-FTIR blood serum spectra for negative (black line) and positive (red line) groups. The vertical lines represents the main vibrational bands contributing to discrimination of groups (see band assignments on Table 2). **(b)** Outliers identification by inspection of $Q^{2}$ residuals (reduced) versus $T^{2}$ Hotelling’s. The outliers were indicated by “*”. Dashed horizontal lines and vertical lines represent confidence limits of 3% (Hotelling’s $T^{2}$) and 97% (*Q* residuals), respectively.

Average FTIR spectra for negative (black line) and positive (red line) classes of samples in the fingerprint spectral window (880–1800 $\hbox {cm}^{-1}$) are shown on Fig. 2. Assignments for the main vibrational bands (vertical lines in Fig. 2a) are presented on Table 2. Proteins, amino acids, and nucleic acids vibrational bands dominate the spectra in accordance with the average chemical composition of the blood serum^31^.

**Table 2:** Assignments for the main vibrational bands of micro-FTIR blood serum (Fig. 2a).

Wavenumber (cm$^{-1}$)	Assignment	References
840–845	Left-handed helix DNA (Z form)	^32^
850–856	$\hbox {C}_{2}^{'}$ endo/anti of deoxyribose in B-form helix conformation	^32^
886	C–C, C–O deoxyribose	^32^
930	Left-handed helix DNA (Z form)	^32^
1030	Stretching C–O ribose	^32^
1080	Ring stretching vibrations in phenylalanine, tryptophan or tyrosine	^32^
1120	Symmetric stretching P–O–C, phosphorylated saccharide residue	^32^
1165	C–O stretching mode of C–OH groups of serine, threonine, tyrosine	^32^
1245	Amide III $\alpha$-helix conformation of proteins	^32^
1315	Amide III of proteins	^32^
1350	Stretching C–O, deformation C–H, deformation N–H	^32^
1400	Symmetric stretching vibration of COO– group of fatty acids and amino acids	^32^
1440	Stretching C–H in polysaccharides, pectin	^32^
1450	Asymmetric $\hbox {CH}_3$ bending in proteins	^32^
1470	$\hbox {CH}_{2}$ bending vibration in lipids and proteins	^32^
1500	In-plane CH bending vibration from the phenyl ring in phenylalanine, tryptophan or tyrosine	^32^
1515–1580	Amide II of proteins	^14,32,33^
1630–1665	$\beta$-sheet structure of Amide I of proteins	^14,32,33^
1683	Unordered random coils and turns of Amide I of proteins	^32,33^
1689–1698	$\beta$-sheet structure of Amide I of proteins	^32,33^
1700–1708	C=O in thymine	^32^
1735	C=O in polysaccharides; new COO– group vibration due to glycated human serum albumin	^32,34,35^
1768–1786	methyl-esterified C=O vibration in IgG COO– group—glycosilation (IgG with sialylate N-glycans)	^33,35–37^

**Figure 3:**
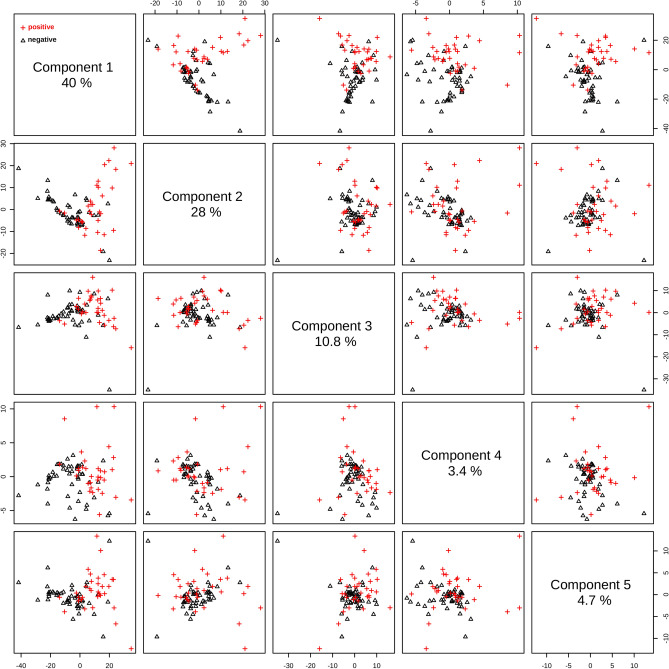
Pairwise score plots for selected PLS-DA components. The explained variance of each component is shown in the corresponding diagonal cell.

Prior data processing and multivariate statistical analysis, a quality check evaluation was performed on raw spectral data to identify anomalous spectra, outliers and/or biased patterns. The PCA was calculated on mean-centered raw data and then *Q* residuals (reduced) versus $T^{2}$-Hotelling’s plot^8,25^ was checked (Fig. 2b) in order to find residuals and scores outliers. Data outside of the confidence limits of 97% for scores and 3% for residuals were considered outliers (indicated by “*” in Fig. 2b) and removed in further analysis. PLS-DA analysis was then performed on processed, normalized, and scaled spectra. The optimal number of factors was determined by cross-validation after inspection of accuracy, $R^{2}$, and $Q^{2}$. Figure 3 presents the pairwise scores up to 5th PC (4.7% of explained variance) for positive and negative groups. At first glance, combinations including components 1 and 2 are prone to discriminate betwenn positive and negative samples.

**Figure 4:**
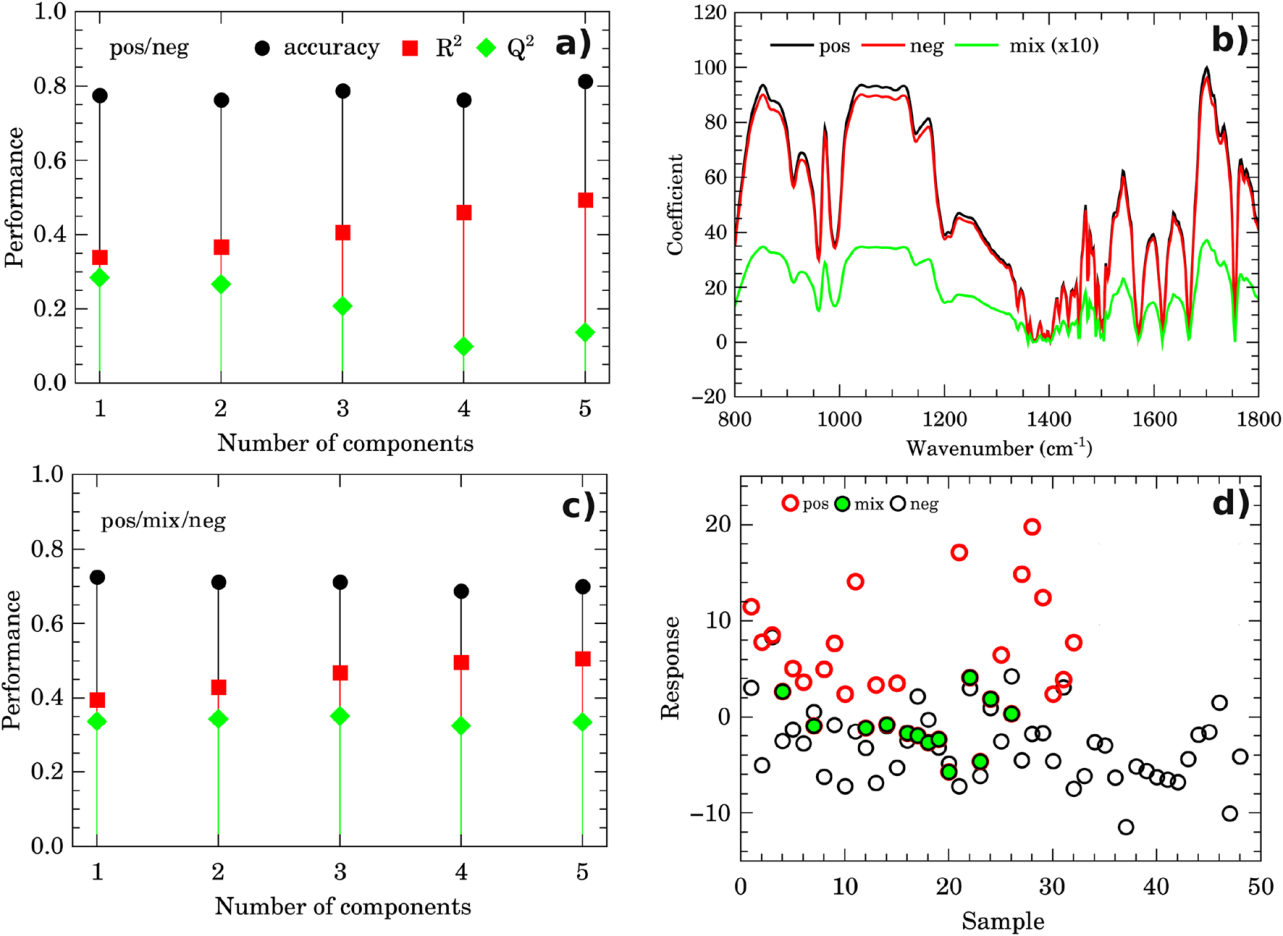
Discrimination performance of micro-FTIR. PLS-DA classification performance using different number of components following accuracy, $R^{2}$ and $Q^{2}$ criteria for two (positive/negative, (**a**) and three (positive/mixture/negative, (**c**) groups. Regression coefficients and calculated response in PLS-DA for sample classes are shown in (**b**,**d**), respectively.

**Figure 5:**
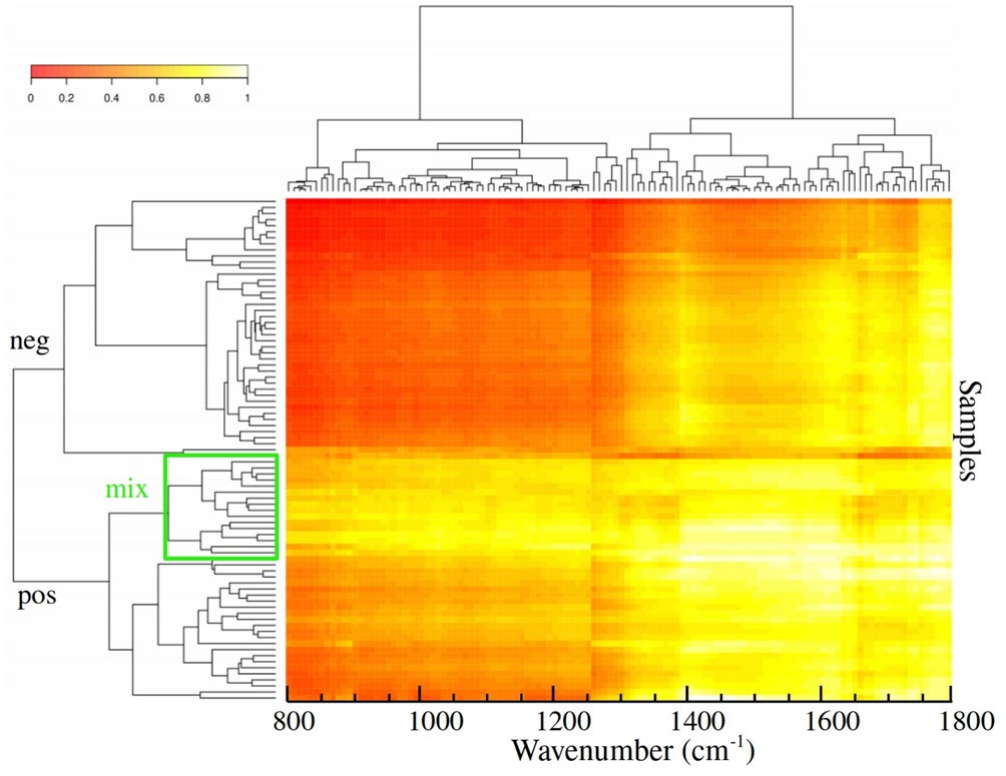
Heatmap for micro-FTIR data. Clustering result shown as a heatmap organized by samples (vertical axis) and wavenumber (horizontal axis). Negative, positive, mix classes grouped into distinct clusters.

The best performance of PLS-DA classification was observed considering 2 components (Fig. 4a). In this case the observed accuracy on groups discrimination was 76% while $R^{2} = 0.39$ and $Q^{2} = 0.34$ (Fig. 4a). Regression coefficients are shown in Fig. 4b). Both curves for discriminating positive and negative groups appeared smooth, showing no random fluctuations around positive and negative values, which would be a symptom of overfitting. However, the coefficients for positive and negative classes appeared superimposed in many spectral windows, indicating greater similarity among them. The calculated response is shown in Fig. 4d). Sensibility and specificity were 53.1% and 87.5%, respectively in this case. Interestingly, there is a distinct subgroup of misclassified samples (labeled here as “mix” group). This subgroup showed a clear spectral signature, as seen in the heatmap in Fig. 5.

**Figure 6:**
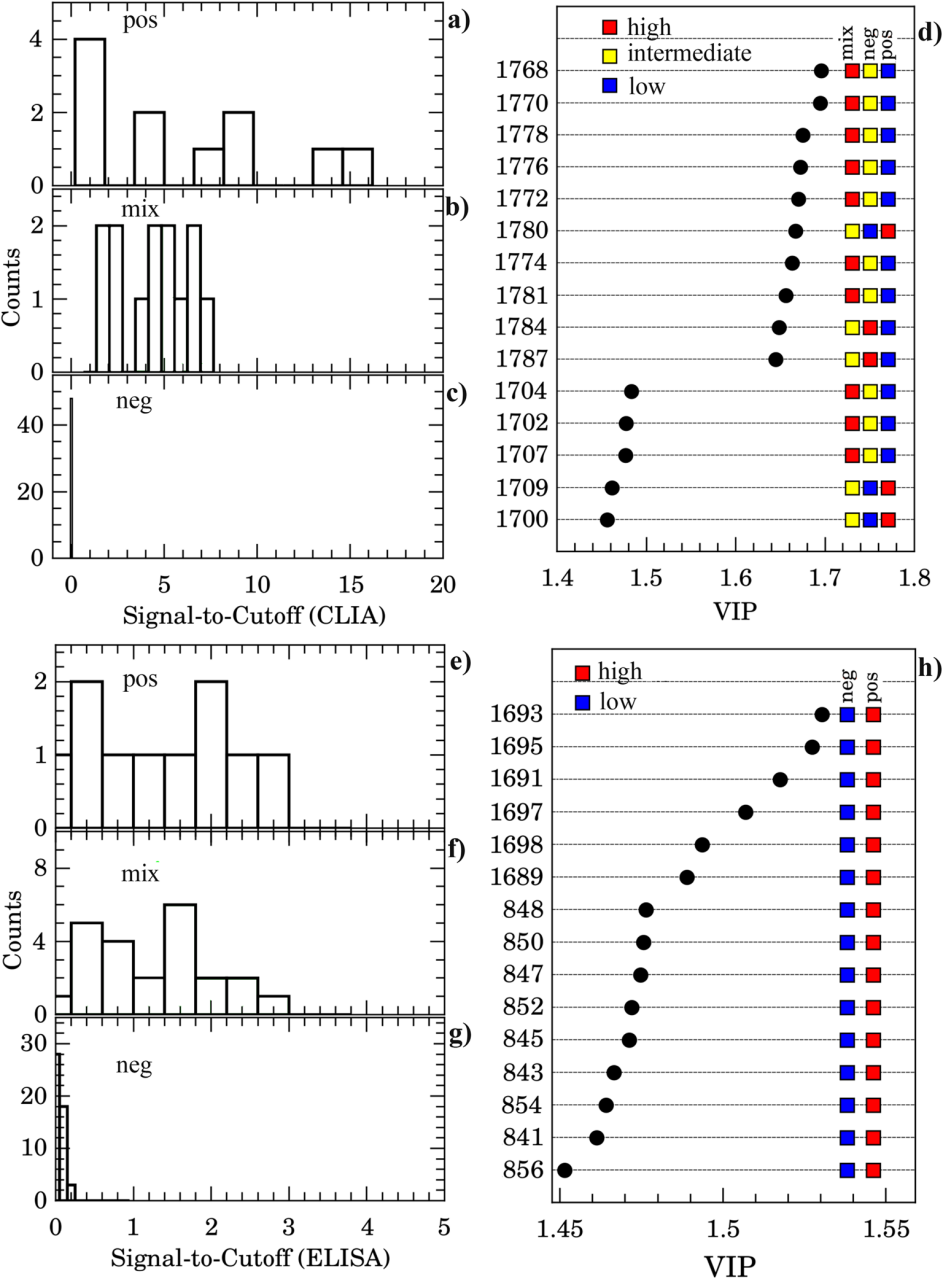
micro-FTIR and CLIA comparison. **(a)**–**(c)** Histograms of signal-to-cutoff data of CLIA IgG antibodies against Sars-Cov-2 for positive (**a**), mix (**b**), and negative (**c**) classes as discriminated by micro-FTIR. **(d)** Important vibrational frequencies (VIP) identified by PLS-DA for three classes classification. **(e)**–**(g)** Histograms of signal-to-cutoff data of ELISA IgG antibodies against Sars-Cov-2 for positive (**e**), mix (**f**), and negative (**g**) classes as discriminated by micro-FTIR. **(h)** VIP for two classes discrimination. Color boxes on the right of **(d)**,**(h)** indicate the relative intensity (high, intermediate and low) of the corresponding band in each group.

The performance of the PLS-DA model with 3 possible groups (positive, negative, and mix) is shown in Fig. 4c). $R^{2}$ and $Q^{2}$ presented a consistent increase of $\sim 0.1$ while the accuracy was around 76%. The coefficient and response obtained are indicated in green in Fig. 4b,d, respectively. Specificity was 87.5% while the detection rate of true positive and true mix increased to 100%. However, one outstanding result was observed contrasting the IgG Signal-to-Cutoff index from CLIA of each positive, mix, and negative groups to FTIR PLS-DA classification data. Histograms in Fig. 6a–c summarize this finding. The positive group presented a broad IgG reactivity index distribution between 1 and 17 while the mix group presented reactivity ranging from 1 to 8. Obviously, the negative group presented a narrow histogram centered on 0.1 value. The outcome from the Z-test ($p<5$%) indicated that these IgG distributions come from distinct populations. Interestingly the histograms of signal-to-cutoff from ELISA (Fig. 6e,f,g) showed no evidence that positive and mixing groups belongs to distinct populations ($p> 5$% for Z test). Thus, the question that arises concerns the characteristics that gives rise to these distinct populations. Important vibrational bands that contribute to discrimination can help find the answer to this question.

The important vibrational frequencies (VIP) identified by PLS-DA are represented in Fig. 6d,h. For positive and negative discrimination (Fig. 6h and Table 2), $\beta$-sheet structure of Amide I of proteins (1689–1698 $\hbox {cm}^{-1}$) and deoxyribose from DNA bands (840–856 $\hbox {cm}^{-1}$) contributed to the greatest over-expression in the positive group. Immunoglobulins are heterodimeric proteins composed of two heavy (H) and two light (L) chains. They can be functionally separated into variable (V) domains that bind antigens and constant (C) domains that drives its functions, such as complement activation or binding to Fc receptors^38^. The basic H2L3 structures consist of the Fc region (fragment, crystallizable) and Fab region (fragment, antigen binding), both composed mainly of $\beta$-pleated sheets^38^.Thus the increased intensity of $\beta$-sheet band in the positive group is completely correlated with antibody recruitment in COVID-19 disease. Likewise, the increase in cellular activity due to Sars-Cov-2 inflammatory response explains the observed increase in DNA band intensity. However, for 3 groups (positive/mix/negative) discrimination, a very narrow set of vibrations confined to 1702–1785 $\hbox {cm}^{-1}$ spectral window is the most important (Fig. 6d). The 1735–1785 $\hbox {cm}^{-1}$ region had been reported as spectral marker for lipids, C$=$O cholesteryl esters, and triglycerides^35^. Besides, the C$=$O bond for the methyl-esterified carbonyl groups also presents a relatively strong absorption peak at 1730–1760 $\hbox {cm}^{-1}$^36^. Also it had been reported that the FTIR spectrum of glycated human serum albumin presents a new peak carbonyl group at $1737\,\hbox {cm}^{-1}$^34^. Also $1739\,\hbox {cm}^{-1}$ and $1781\,\hbox {cm}^{-1}$ bands were reported as predictors for classifying anti-neutrophil cytoplasmic antibodies in sera samples^39^

**Figure 7:**
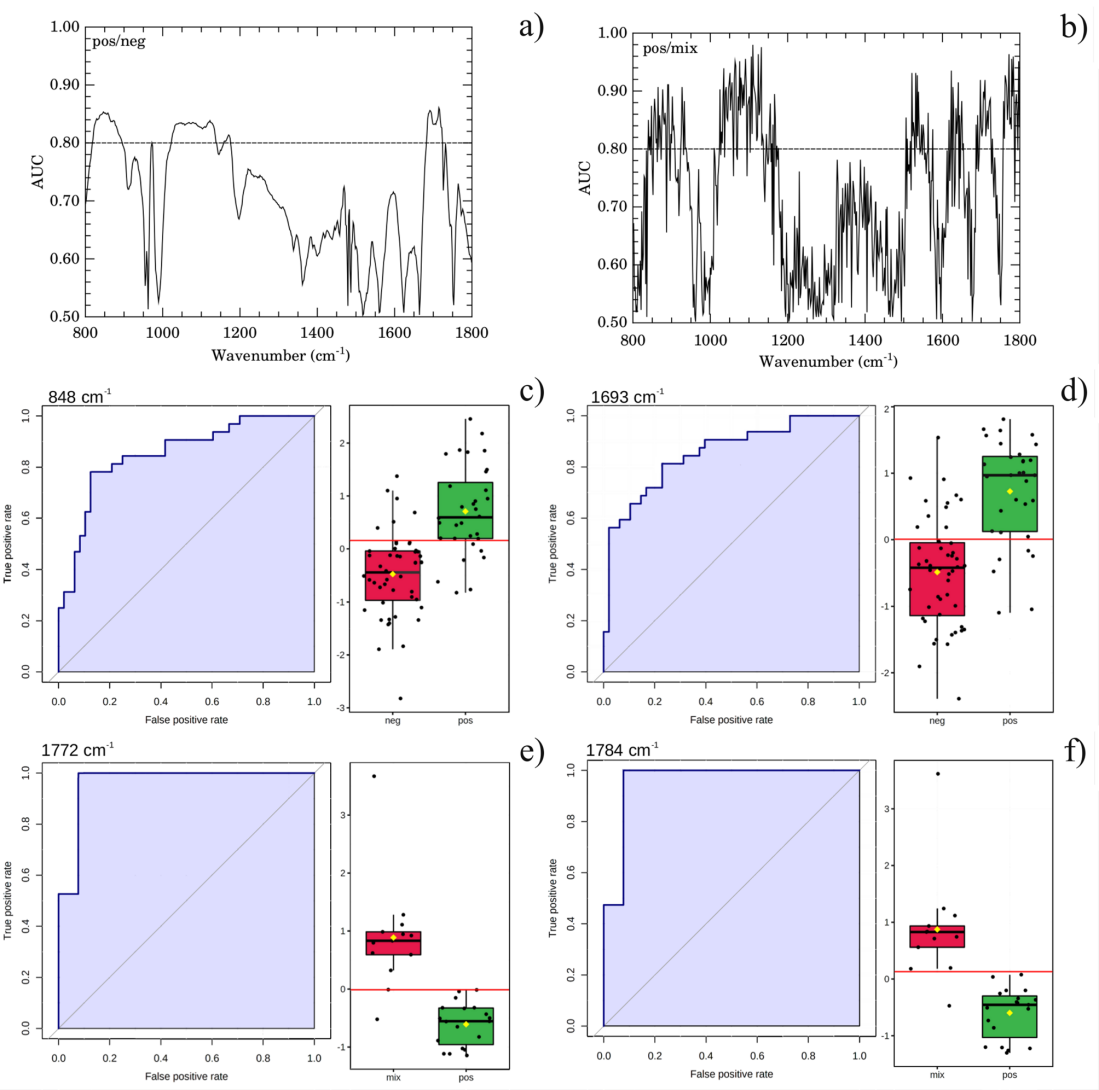
Diagnostic performance of micro-FTIR. Area under receiver operating characteristic (*AUC*) against wavenumber showing those bands with excellent discriminating power ($AUC > 0.80$, dashed horizontal line) for positive/negative (**a**) and positive/mix (**b**) classes. Selected representative curves of receiver operating characteristic (ROC) and corresponding classification box-plot of the intensity of the left-handed helix DNA (Z form) ($848\,\hbox {cm}^{-1}$, **c**), $\beta$-sheet structure of Amide I of proteins ($1693\,\hbox {cm}^{-1}$, **d**), C=O in IgG carbonyl group (1772 and $1784\,\hbox {cm}^{-1}$ in (**e**,**f**), respectively) bands. The horizontal red line is the threshold for classification.

.

In our case bands in 1702–1785 $\hbox {cm}^{-1}$ spectral window could be grouped in two classes: (1) those overexpressed in the mix group and subexpressed in the positive group which are both related to IgG glycosylation and (2) those overexpressed in the positive group and underexpressed in the mix group which are both related to thymine. As long as we can argue that the mix group relates to a COVID-19 sub-population with highly glycoyilated IgG. As all patients in the present study were mild or oligosymptomatic, the above feature is in agreement with the Chakraborty et al. findings^3^ which proposed that patients with severe COVID-19 are more likely to have IgG1 with afucosylated Fc glycans as signature. It is well reported that IgG glycosylation can determine whether an antibody glycoform is pro-inflammatory (such as IgG with galactose-deficient N-glycans) or anti-inflammatory (such as IgG with sialylate N-glycans)^37^. Since 1768–1786 $\hbox {cm}^{-1}$ region is related to methyl-esterified carbonyl vibration we argue that this band is related to C=O on sialic acid on IgG with sialylate N-glycans. One important point to clarify is the difference between CLIA and ELISA distribution of Signal-to-cutoff ratio (Fig. 6). It appears that the fluorescence yield of murine monoclonal anti-human IgG anti-body complexed to HRP of CLIA is dependent on the fucosylation of the Fc tail of human IgG. This point needs to be explored in additional experiments.

Concerning the diagnostic performance we mention that spectral windows at 820–890, 1025–1180, and 1685–1727 $\hbox {cm}^{-1}$ presented excellent capability for discriminate negative and positive classes (AUC = 0.80–0.86, Fig. 7a). The positive and mix classes were also discriminated in excellent grade (AUC = 0.80–0.98) in spectral windows 850–940, 1015–1170, 1500–1570, 1612–1652, 1695–1735, 1755–1790 $\hbox {cm}^{-1}$ (Fig. 7b). This curve is comparatively nosiest respect to the positive/negative case due to the low-N (only 30% of data) in this case. The representative ROC curves for negative and positive (Fig. 7c,d) and mixing and positive (Fig. 7e,f) classes with highest *AUC* are also shown. The ROC curves for positive/negative (Fig. 7c,d) have binormal curve shapes while mixing/positive ones have straight line shapes due to low-N. The classification boxplots for two discriminating classes presented a significant statistical difference among groups ($p < 0.05$ for t-Student test).

### Micro-FTIR reflectance compared to other methods

At this point it is important to compare our results to previous reports concerning blood testing for COVID-19. The usage of ATR-FTIR for analysis of plasma from COVID-19 patients have been recently reported in literature. This technique shares a great similarity with our methodology and for this reason a detailed comparison is important.

Banerjee et al.^40^ investigated a cohort of 160 clinicopathologically confirmed SARS-CoV-2 patients using ATR-FTIR. They proposed a plasma processing with 75% ethanol v/v for virus inactivation followed by vortexing and drying over ATR-crystal. The reported *AUC* was 0.851 when considering the set of spectral and clinical (age, sex, diabetes mellitus, and hypertension) data. The discriminating performance of the spectral data alone was not reported. Zhang et al.^41^ investigated by ATR-FTIR blood samples from 20 healthy donors and 76 patients, of which 41 were confirmed with COVID-19, 15 had respiratory viral infections caused by influenza A/B or respiratory syncytial virus (RSV), and 20 were with inflammation-related diseases. In this case the *AUC* for COVID-19 discrimination was reported to be 0.9947. To the best of our knowledge only these two exciting articles reports COVID-19 diagnosis using ATR-FTIR on blood or plasma samples. In spite of diverse sample preparation method and amount of individuals included in the cohort, our *AUC* is very similar to that found by Banerjee et al. and Zhang et al. The key differences between our approach and the ATR-FTIR relies on waiting time for result outcome, amount of samples which would be tested by row, and COVID-19 fatality capability prediction since we explored in deep the biochemical pieces of information in the FTIR spectra. For spectrum acquisition on ATR-FTIR a single drop of sample need the deposited and dried over the surface of ATR crystal. Then only a single patient could be tested at once. This process demands usually > 5 h^35^. It is not well reported whether the proteins degradation over this time would impact on quality of diagnosis. Moreover the morphological characteristics of the deposited bio-film (heterogeneity, size, wettability, dilution, among others) is dependent of drying conditions as relative moisture and temperature. The quality of spectrum (artifacts, reproducibility) is very sensitive to the morphology of the bio-film. These points impose challenges to the standardization of the method^35^. On the other hand, our approach enables the preparation of drops of several patients over the substrate. Over an equivalent area of a microplate of 96 wells it is possible the deposition of 500 droplets. Using the microscope re-positioning the IR beam over each patient drop is relatively easy which increases the scale of testing. Banerjee et al. reported the fatality prediction only when considering in the statistical analysis a larger set of data of different kinds as spectra and clinical pieces of information which in some aspect would represent a bias in the statistical test. We were able to discriminate 3 possible groups in an independent way of validation. The clinical data were used to compare and discuss the data but not as input.

Other comparison is with the gold standard method for COVID-19 testing ELISA and also with CLIA method. Both were used to validate our results. Jagtap et al.^42^ evaluated the performance of spike protein antigens for SARS-CoV-2 serology compared four spike proteins: RBD, S1, S2 and a stabilized spike trimer (ST). They used indirect ELISA in serum from COVID-19 patients and pre-2020 samples. ROC curves indicate that ST is the best candidate for serological testing with the highest *AUC* ($AUC=0.94$). AUC for IgG was 0.934. Huang et al.^43^ compared the serological test of SARS-CoV-2 using ELISA test and reported that $AUC(IgM)=0.812$ while $AUC(IgG+IgM)=0.983$. The reported *AUC* for CLIA anti-Sars-CoV-2 test include 0.846^44^ , 0.9143^45^, 0.98^46^, and 0.831^44^. Thus we can conclude that the performance of our micro-FTIR approach is in the same level of ELISA and CLIA.

However as summarized by Liu and Rusling^47^, in general the nucleic acid-based tests against COVID-19 have reported high false-negative rates which are dependent of the day of the symptom (38% on the day, 20% 3 days, and 66% 16 days, after of symptom onset, respectively). Factors such as sampling time, viral mutation, inadequate handling, improper storage, transportation of samples, among others had been cited to influence the result. The requirements to perform these tests evolve high workload, skilled personnel for testing and sample collection, special reagent kits, costly centralized infrastructure with specific equipment for process and measured the sample signal, and professional bio safety level (BSL)-2 lab^47^ imposing elevated costs, relatively longer time to delivery of results (2–3 h) and dependence of international chain of suppliers. This last aspect would be disrupted during the pandemic period. One important advantage of the micro-FTIR method is the minimal need of sample preparation which impacts lower cost per testing and relative independence of external suppliers. The time elapsed pipetting , drying, acquiring spectra is typically 10 min. Another aspect to mention is that ELISA and CLIA tests are able to detect a single protein at once. Nevertheless FTIR has multiplexing advantage enabling comparison of several metabolites which increases the quality of prognosis being a suitable tool for precision medicine^48^. Our method enables in principle to probe 500 samples per row while ELISA and CLIA usually are limited to 96 samples. Table 3 summarizes the comparison between all techniques.

**Table 3:** Parameters for maintenance, management, operating, and performance of the main anti-Sars-Cov-2 clinical tests ELISA and CLIA compared to the ATR-FTIR and micro-FTIR.

Parameter	Micro-FTIR	ATR-FTIR	ELISA	CLIA
Sample preparation	Minimal	Minimal	Complex	Complex
Waiting time for result	$$<10$$ min	$$> 5$$ h	2–3 h	2–3 h
Laboratory equipment requirements	Intermediate	Intermediate	High	High
Specialization of human resources for usage	Intermediate	Intermediate	High	High
*AUC*	$$> 0.85$$	$$> 0.85$$	$$> 0.85$$	$$> 0.85$$
Multiplexing capability	Yes	Yes	No	No
COVID-19 fatality prediction	Yes	Depends	No	No
Reagents	Free	Free	Expensive	Expensive
Cost of single test (US$)	1–10	1–10	50–100	50–100
Cost of main equipment for testing (US$)	20,000–50,000	20,000–50,000	10,000–30,000	10,000–30,000
Amount of samples tested by row	$$> 500$$	1	96	96
Dependence of international logistic and supply chains	Weak	Weak	Strong	Strong
Operator dependence level of reproducibility of outcome	Intermediate	Intermediate	High	High

## Conclusions

Our results showed that micro-FTIR was able to probe key structural aspects of serum IgG from COVID-19 volunteers. The 1702–1785 $\hbox {cm}^{-1}$ spectral window is a spectral marker of the degree of IgG glycosylation, allowing to probe distinctive sub-populations of COVID-19 patients, depending on their degree of severity. Furthermore, the $\beta$-sheet structure of Amide I of proteins (1689–1698 $\hbox {cm}^{-1}$) and deoxyribose from DNA (840–856 $\hbox {cm}^{-1}$) bands presented most significant contributions to positive and negative discrimination with a specificity of 87.5% and sensibility of 100%. The computed *AUC* was comparable to the ELISA, CLIA, and other ATR-FTIR methods ($AUC > 0.85$). In summary, overall discrimination of healthy and COVID-19 individuals and severity prediction could also be potentially implemented using micro-FTIR reflectance spectroscopy on blood serum samples due to direct probe of glycosylation degree of IgG. However, experimental efforts need be devoted to isolate immunoglobulins molecules from plasma samples from healthy and COVID-19-ill patients and characterize their spectral properties what would give detailed clues on IgG glycosilation and other structural changes related to pathology.
